# The lichen secondary metabolite lichesterinic acid exhibits antibiofilm activity against fungal pathogens

**DOI:** 10.3389/fcimb.2025.1730365

**Published:** 2026-01-12

**Authors:** Martin N. Odabas, Katharina Kainz, Ingo Weinberger, Kerstin Schloffer, Sabrina Riedl, Marylène Chollet-Krugler, Dagmar Zweytick, Joël Boustie, Frank Madeo, Didac Carmona-Gutierrez

**Affiliations:** 1 Institute of Molecular Biosciences, NAWI Graz, University of Graz, Graz, Austria; 2 BioHealth Graz, Graz, Austria; 3 BioTechMed Graz, Graz, Austria; 4 CNRS, Institut des Sciences Chimiques de Rennes—UMR 6226, Univ Rennes, Rennes, France

**Keywords:** antimycotics, biofilm eradication, biofilm inhibition, *Candida albicans*, *Candida glabrata*, drug, yeast, resistance

## Abstract

Lichens are well known for producing unique secondary metabolites, some of which have been shown to exhibit medically relevant bioactivities, including antimicrobial effects. With the increasing prevalence of fungal infections and the growing resistance to commonly used antimycotics, there is an urgent need for new antifungal agents, especially for aged and/or immunocompromised individuals. In this study, we screened a collection of lichen-derived metabolites for antifungal properties in two medically relevant fungal pathogens, *Candida albicans* and *Nakaseomyces glabratus*. Several compounds exhibited inhibitory effects against planktonic cells and/or biofilm formation in at least one of these species. Notably, two related paraconic acids demonstrated the strongest activity against biofilms, structures that contribute significantly to antifungal resistance. Among them, lichesterinic acid was the most effective in disrupting pre-formed biofilms and preventing biofilm formation, key challenges in clinical mycology. Importantly, lichesterinic acid showed moderate tolerability in human cells. Furthermore, lichesterinic acid displayed antifungal efficacy in an *in vivo* model of fungal infection, supporting its potential for therapeutic development. These findings highlight lichen-derived metabolites, particularly paraconic acids, as promising candidates for new antifungal therapies targeting resistant and biofilm-associated fungal infections.

## Introduction

In the 21st century, infectious diseases remain a major health issue and one of the leading causes of death. Among them, fungal infections (FIs) were long underestimated in terms of severity. Although fungal pathogens are widely recognized for causing superficial infections of the skin and nails, the serious morbidity and mortality linked to invasive FIs has historically received less attention. In recent years, however, this perception has shifted due to the alarming rise in (multi-)resistant strains, which has led to significantly increased clinical burden and socioeconomic consequences (Lockhart and Guarner, 2019). While invasive FIs are rare in healthy individuals (Robert and Casadevall, 2009), they pose a threat for susceptible people like elderly, critically ill and immunocompromised persons and are associated with drastic mortality rates (Enoch et al., 2017). Of note, this risk group is continuously growing due to the overall increase of lifespan and development of new immune-modulating therapeutics for cancer and other diseases (Friedman and Schwartz, 2019; Lockhart and Guarner, 2019). The most recent estimates suggest that invasive FIs cause 3.8 million deaths worldwide each year (Denning, 2024). Among the four species listed in the most critical group are two *Candida* species (*C. auris* and *C. albicans*). *C. albicans* remains the predominant *Candida* species in most regions worldwide (Vallabhaneni et al., 2016), while non-albicans *Candida* (NAC) species such as *C. auris*, *C. dubliniensis*, *Pichia kudriavzevii* (formerly classified as *C. krusei*), or *Nakaseomyces glabratus* (formerly classified as *C. glabrata*) are of increasing clinical relevance due to their absent or reduced susceptibility to common antifungals (Deorukhkar et al., 2014; Beardsley et al., 2024).

Current antifungal therapy is severely limited, with only four major classes of antifungal agents available for clinical use (Hossain et al., 2022). This restricted arsenal is further compromised by the growing problem of resistance, which includes both intrinsic resistance and acquired resistance resulting from prolonged antifungal exposure. A major driver of this resistance crisis is the widespread, and often indiscriminate, use of antifungal agents—not only in clinical settings as prophylactic treatments but also in agriculture and animal husbandry, where their extensive application has created an environmental reservoir of drug-resistant pathogens (Perlin et al., 2017). Adding to this challenge is the ability of many fungal pathogens like *Candida* spp. to form biofilms. Biofilms are complex, structured cell communities that adhere to surfaces and are encased in an extracellular matrix, which protects them from many physical as well as chemical influences. Heterogenic metabolic activity as well as up-regulation of efflux pumps give these communities additional protection against chemical attacks and makes them less susceptible to antifungals compared to (free-floating) planktonic cells (Fanning and Mitchell, 2012), further complicating treatment. In fact, the effectiveness of many commonly used azoles against biofilm-associated *Candida* spp. infections is notably limited and species-dependent (Kaur and Nobile, 2023), often requiring the use of alternative agents that are both more expensive and associated with greater toxicity. Notably, single cells can detach from the protective environment of the biofilm and start new foci of infections at distal sites, making biofilms also a source for persistent and recurring FIs (Wall et al., 2019). Aside from host surfaces, biofilms can also form on implantable medical devices, like intravascular catheters (Ramage et al., 2006), and release of cells from a biofilm-infected catheter can lead to disseminated or blood-stream infections (Uppuluri et al., 2010). Together, these factors highlight the urgent need for novel pharmacological strategies that can bypass existing resistance mechanisms and, ideally, exert anti-biofilm activity.

Lichens are symbiotic associations between fungi (typically ascomycetes) and photosynthetic partners (algae or cyanobacteria). They are known to produce diverse secondary metabolites, most of which exclusively occur in lichens (Ranković and Kosanić, 2015). These compounds are typically synthesized by the fungal partner via diverse pathways like the polymalonate, shikimic acid and mevalonic acid pathways (Boustie and Grube, 2005; Singh et al., 2025) and play distinct ecological and biological roles, such as UV protection, defense against herbivores, antimicrobial activity, and communication with symbiotic partners. Among them, a number of metabolites with medicinal potential have been identified, including such with antibacterial, antiviral, anti-inflammatory, and anti-proliferative effects (Boustie and Grube, 2005). In addition, several lichen-derived extracts and compounds have been shown to exert antifungal activity, including against *Candida* spp (Millot et al., 2017; Poulsen-Silva et al., 2025). However, the potential of lichen secondary metabolites as a source of novel antimycotics remains largely underexplored.

Based on these thoughts, we set out to screen a library of 68 unique lichen-derived compounds (LDCs) for their activity against *C. albicans* and *N. glabratus*. We selected these two species to take into account the spectrum of clinically relevant *Candida* pathogens: *C. albicans* as the most prominent cause of candidiasis and *N. glabratus* as a representative of non-albicans species. Importantly, both species are among the most relevant biofilm-forming fungal pathogens with increasing rates of intrinsic or acquired resistance to common antifungals. We assessed different virulence aspects, including efficacy against planktonic cells, biofilm inhibition and eradication of pre-existing biofilms, identifying the paraconic acid (+)-lichesterinic acid (LIA) as a top hit with both *in vitro* and *in vivo* effects. Altogether, we were able to construct a screen-to-hit pipeline that has delivered novel candidates with pharmacological potential against fungal biofilms.

## Materials and methods

### Lichen-derived compounds

Lichen secondary metabolites were mainly obtained as gifts from the Huneck’ chemical library (Botanical Museum in Berlin), stored in the lab of Pharmacognosy of the Faculty of Pharmacy in Rennes (Institute of Chemical Sciences). For most compounds, information concerning isolation of these compounds from lichens or obtention as lichen compound derivatives can be retrieved (Huneck and Yoshimura, 1996; Desmarets et al., 2023). (+)-Lichesterinic acid (LIA) was obtained by synthesis according to Sweidan et al. (Sweidan et al., 2016). Note that diterpene compounds are not usually reported from lichen; however, the diterpene compounds 1, 32 and 35 in this screen have been recently described to occur in lichens (De Angelis et al., 2003; Kerboua et al., 2021; Koopaie et al., 2023).

Crystallized LDCs were dissolved in dimethyl sulfoxide (DMSO) to a concentration of 25 mM directly before first usage and stored at -20°C for potential subsequent validation analyses. In some cases, the compounds were not completely soluble at this concentration and were further diluted with DMSO to reach total solubilization. The highest tested concentration for each LDC can be found in **Supplementary Table 1**. All assays were conducted with freshly dissolved compounds except for the low-throughput BFI assays in *N. glabratus* of LDCs 48, 51, 55, 56, 59 and 60 for screen hit validation. Fresh stocks were no longer available, and frozen stocks were used instead.

### *Candida* strains and growth conditions

*C. albicans* SC5314 wild type (WT) was used for all antifungal experiments. *N. glabratus* BG2 (WT) was only used for the screen and validation of LDCs as well as for biofilm eradication experiments. *C. dubliniensis* IHEM 14280 (WT) and *Pichia kudriavzevii* IHEM 6104 (WT) were only used for complementary experiments with LIA. All growth media were prepared with ddH_2_O. YPD (10 g/l yeast extract, 20 g/l peptone, 20 g/l D-glucose) and YPD agar plates (YPD + 20 g/l agar) were subsequently autoclaved (25 min, 121°C, 210 kPa). RPMI media (10.4 g/l RPMI-1640 powder (Sigma), 34.53 g/l MOPS, pH adjusted to 7.0 with NaOH) was sterile filtered and stored at 4°C.

*C. albicans* and *N. glabratus* were streaked on YPD agar plates and incubated at 37°C for 24 h. For each experiment overnight cultures (ONCs) of *C. albicans* and *N. glabratus* were inoculated with material from YPD agar plates (not older than 2 weeks) in 3 ml YPD in glass eprouvettes and incubated at 30°C at 170 rpm shaking for 14 h – 18 h.

For antifungal susceptibility testing, *C. albicans* and *N. glabratus* were grown in RPMI media at 37°C to simulate human blood conditions. For experiments on planktonic cells, they were grown in 96-well flat bottom plates (Greiner Bio-One, Austria) which were sealed with gas-permeable adhesive membranes (Excel Scientific) and closed with a lid. Biofilms were grown in 96-well tissue culture test plates (U-bottom, TPP, Switzerland) which were closed with the supplied lids. The rim wells of either plate were not used because they are prone to drying out. They were filled with 100 µl of sterile ddH_2_O instead to avoid evaporation.

### Minimal inhibitory concentration

The minimal inhibitory concentration (MIC) of LDCs in *C. albicans* and *N. glabratus* was determined following the standard Clinical and Laboratory Standards Institute (CLSI) protocol M27 for yeast strains (CLSI, 2017) with some modifications. *C. albicans* and *N. glabratus*, were both treated with two-fold serial dilutions of LDCs. For 25 mM stocks of LDCs the tested final concentrations were 250 µM, 125 µM, 62.5 µM, 31.3 µM, 15.6 µM, 7.8 µM, 3.9 µM and 2.0 µM. Serial dilutions were done with DMSO and then further diluted 1:10 with RPMI. 10 µl of those dilutions were then added to the wells of 96-well flat bottom plates. In some cases, this initial dilution resulted in visible precipitation at the 1–2 highest concentrations; in these instances, particular care was taken to thoroughly resuspend the sample and to accurately apply 10 µl. ONCs were prepared in 3 ml YPD and incubated at 30°C and 170 rpm for 14-18 h. Fresh RPMI was inoculated with ONCs to an OD_600_ of 0.001 (appr. 1x10^4^ cells/ml) and vortexed thoroughly. 90 µl of the prepared inoculum were then added to each well of 96-well flat bottom plates already containing the diluted compounds. After mixing, the plates were sealed with gas-permeable adhesive membranes, covered with a lid and incubated without shaking at 37°C for 48 h. Each plate contained a two-fold serial dilution of amphotericin B (5 µM - 0.31 µM) as antifungal control and six wells with 1% DMSO as growth controls. OD_490_ was measured after 24 h and 48 ± 1 h with a plate reader (Glomax multi+ detection system, Promega). To analyze growth capacity measured OD_490_ values were then normalized to the mean of corresponding growth controls. Dose-response curves were generated and, if possible, MIC_50_ values (= concentration at which growth was inhibited by 50% compared to untreated control cells) were calculated (GraphPad Prism 8).

### Biofilm inhibitory concentration

To assess biofilm inhibitory concentrations (BIC) in *C. albicans* and *N. glabratus*, both were grown under biofilm-inducing conditions and treated with two-fold serial dilutions of LDCs. Assays were performed as described by Delattin et al. (Delattin et al., 2014) with some modifications. Serial dilutions of the compounds were prepared as previously described (see above) and 10 µl from each of these conditions were then loaded onto the wells of 96-well tissue culture test plates. Concentration of compounds and controls were the same as in MIC experiments. RPMI was inoculated to an OD_600_ of 0.1 with ONCs and 90 µl of the culture were then added to each well of the U-bottom plates. Plates were then incubated for 1 h at 37°C to allow adherence of the cells. The supernatants with non-adherent cells were then removed and the adherent cells were gently washed with 100 µl PBS pH 7.4 (80 g/l NaCl, 2 g/l KCl, 14.4 g/l Na_2_HPO_4_, 2.4 g/l KH_2_PO_4_). 100 µl of fresh RPMI with compounds were added and the plates were incubated at 37°C.

After 24 ± 1 h of incubation, inhibition of the biofilms was assessed through measurement of metabolic activity. Biofilms of *C. albicans* were stained with the resazurin-based redox dye cell titer blue^®^ (CTB; Promega, Germany) and biofilms of *N. glabratus* were stained with the tetrazolium salt XTT (ABCR, Germany). For both staining procedures, supernatants with non-adherent cells were removed and biofilms were gently washed with 100 µl PBS pH 7.4. Biofilms of *C. albicans* were then stained with 100 µl CTB staining solution (CTB diluted 1:100 in PBS pH 7.4) and biofilms of *N. glabratus* were stained with 100 µL XTT staining solution (0.25 mg/mL XTT + 0.1 µM menadione in PBS pH 7.4). The plates were then incubated at 37°C for 1 h in the dark. For XTT staining in the low throughput validation round as well as for biofilm eradication, biofilms were stained with 120 µl of XTT staining solution instead and after incubation, 100 µl of the supernatant were transferred into fresh 96-well flat bottom plates for measurements. For biofilms stained with CTB, fluorescence was measured by excitation with 525 nm and emission at 580 nm – 640 nm. For biofilms stained with XTT, OD_490_ was measured. All measurements were done with a plate reader (Glomax multi+ detection system, Promega). To assess viability of cells in biofilms measured values were normalized to the mean of corresponding growth controls. Dose-response curves were generated and, if possible, BIC_50_ values (= concentration at which metabolic activity in biofilms was inhibited by 50% compared to untreated controls) were calculated (GraphPad Prism 8).

### Biofilm eradication

To assess effects of compounds against pre-grown biofilms of *C. albicans* and *N. glabratus*, biofilm eradication (BFE) measurements were conducted. Assays were performed as described by Vriens et al. (Vriens et al., 2015) with some modifications. RPMI was inoculated to an OD_600_ of 0.1 with an ONC and 100 µl aliquots of the culture were then added to wells of 96-well tissue culture test plates. The plates were then incubated for 1 h at 37°C to allow adherence of the cells. The supernatants were removed and adherent cells were gently washed with 100 µl PBS pH 7.4 to remove non-adherent/planktonic cells. 100 µl of fresh RPMI, preheated to 37°C, were added to the wells and plates were incubated at 37°C. After 24 h the supernatants were removed, the biofilms were gently washed with 100 µl PBS to remove non-adherent/planktonic cells and two-fold serial dilutions of compounds mixed with RPMI were added. The plates were then incubated at 37°C. After 24 h supernatants were removed from the incubated U-bottom plates and the wells were gently washed with 100 µl PBS pH 7.4 to remove non-adherent/planktonic cells. Biofilms were stained, incubated and measured as described for the assessment of BIC. To assess viability of cells in biofilms measured values were normalized to the mean of corresponding growth controls. Dose-response curves were generated and, if possible, BEC_50_ values (= concentration at which metabolic activity in pre-grown biofilms was inhibited by 50% compared to untreated controls) were calculated (GraphPad Prism 8).

### Human cell viability assay

To assess toxicity of LDCs against human cells, viability assays were conducted with Normal Human Dermal Fibroblasts (NHDF) from an adult donor (PromoCell). Assays were conducted in Fibroblast Growth Medium 2 (PromoCell). DMSO content was 0.5% for both LDC-treated cells and untreated controls. To determine the viability of LDC-treated cells, CellTiter-Glo^®^ 3D Cell Viability Assay (Promega G9682, Madison, WI, USA) was used. The assays were performed according to the manufacturer’s guidelines. Cells were harvested at confluency of approximately 90% and diluted to a concentration of 10^5^ cells/100 µl. Subsequently, 100 µl aliquots were dispensed into 96-well white, clear bottom 96-multiwell plates (Fisher Scientific GmbH, Austria). The measurements were performed with the GloMax^®^ Discover Microplate Reader (Promega, Madison, WI, USA) at room temperature.

Cells were grown overnight and two-fold serial dilutions of LDCs were added to a final concentration of 250 µM, 125 µM, 62.5 µM, 31.3 µM, 15.6 µM, 7.8 µM and 3.9 µM. After LDC addition, they were incubated for 24 h at 37°C and 5% CO2. After 24 h equal volume of the CellTiter-Glo-Reagent (100 µl) was added to each well of the 96-well plate. The plate was inserted into the measurement device where the plate was shaken vigorously for 5 min, incubated for 25 min and the luminescence was recorded.

CellTiter-Glo^®^ 3D Cell Viability Assay (Promega G9682, Madison, WI, USA) was used to determine the cell viability in 2D *in vitro* cell culture. The luminescence measurements were performed with the assay reagent (Ultra-Glo™ rLuciferase) to determine ATP, which indicates viability of the cells and generates a detectable luminescence readout. For 100% toxicity level the cells were treated with 2 µl Triton-X-100 (10%) representing 0% of cell viability (V_0_). Cells in the absence of LDCs and Triton were a negative control (V_100_).

The viability of each well was calculated from the percentage of viable cells without LDCs and in the presence of LDCs (V_x_) with the following equation: 


$$\%viability=100*\frac{\left(V_{x}-\;V_{0}\right)}{\left(V_{100}-\;V_{0}\right)}$$

### Filamentation

Filamentation assays on solid media were performed as described by Böttcher et al. (Böttcher et al., 2020). Cells from an ONC of *C. albicans* were harvested and washed with an equal volume of PBS. Cells were then diluted in PBS to an OD_600_ of 0.5 and 5 µl were spotted onto Spider (1% nutrient broth, 1% mannitol, 0.2% dipotassium phosphate and 1.35% agar), Spider D (1% nutrient broth, 0.5% glucose, 0.2% dipotassium phosphate and 1.35% agar) and RPMI (RPMI media with 2% agar) agar plates. Plates were incubated at 37°C and colony morphology was documented on days 3, 5 and 7.

### Time-kill assay

Potential fungicidal effects against *C. albicans* were investigated by time-kill and antifungal carryover assays. Both assays were performed as described by Klepser et al. with minor modifications (Klepser et al., 1997). Flasks with 10 ml of RPMI media were inoculated to an OD_600_ of 0.001 with an ONC of *C. albicans* and treated with either 160 µM, 80 µM or 40 µM of compound. DMSO was used for untreated controls and DMSO content was 1% for all flasks. Three replicate flasks were done for each treatment and controls. Flasks were incubated at 35°C at 150 rpm shaking for 24 h. Samples were taken, serially diluted in PBS and plated on YPD agar plates after 0, 2, 4, 6, 8, 12 and 24 h. The 0 h timepoint was sampled before addition of treatments. Plates were incubated at 30°C for 48 h for colony count determination. Data was plotted as log CFU/ml for each time point.

Antifungal carryover was assayed for all treatment concentrations of the time-kill assay in three replicates by plating 3-5x10^2^ freshly diluted cells from an ONC of *C. albicans* onto YPD agar plates immediately after treatment. Plates were incubated at 30°C for 48 h for colony count determination. Antifungal carryover was defined as negative when the difference between control and treatment was ≤ 25%.

### Acute toxicity

YPD was inoculated to an OD_600_ of 0.1 and incubated at 30°C at 150 rpm shaking until an OD_600_ of 0.3-0.5 was reached. Three samples were taken for timepoint 0 and the culture was then split into 10 ml aliquots for treatments and controls. Flasks were treated with either 80 µM, 60 µM or 40 µM of compound. DMSO was used for untreated controls and DMSO content was 1% for all flasks. Three replicate flasks were done for each treatment and controls. Flasks were incubated at 30°C at 150 rpm shaking for 2 h. For plating, samples were taken, serially diluted in PBS and plated on YPD agar plates after 0, 30 and 60 min and incubated at 30°C for 2 days for colony count determination. Data was plotted as log CFU/ml for each time point.

Cell death was assessed by propidium iodide (PI) staining. 1.25 ml of culture were sampled at the same time points as for plating. Samples were centrifuged at 10,000 g for 1 min and supernatant was removed. Cells were then resuspended in 500 µl PI staining solution containing 100 ng/ml of PI (Sigma, Germany) in PBS pH 7.4 and incubated for 15 min in the dark. Cells were then centrifuged again, the supernatant was removed and cells were resuspended in 250 µl of PBS. 200 µl of cell suspensions were transferred into 96-well U-bottom plates (Greiner Bio-One, Austria) and analyzed by flow cytometry (LSRII Fortessa, BD). Fluorescence (excitation: 561 nm, emission: 670/30 nm) was quantified at the single cell level, for each sample, 30,000 cells were analyzed with BD FACSDiva software.

### *Caenorhabditis elegans* fungal infection model

*In vivo* testing of LIA in the *C. elegans* infection model was done as described by Breger et al. (Breger et al., 2007) with some modifications. To synchronize nematodes, eggs of the *C. elegans* Δ*glp-4* Δs*ek-1* strain were collected by bleaching as described by Porta-de-la-Riva et al. (Porta-de-la-Riva et al., 2012), placed on NGM (nematode growth medium) plates (2.5 g/L Bacto Peptone, 3 g/L NaCl, 17 g/L agar, supplemented with 5 mg/L cholesterol, 1 mM CaCl_2_, 1 mM MgSO_4_ and 25 mM KPO_4_ buffer pH 6.0) agar plates seeded with *Escherichia coli* OP50 and incubated. Nematodes were then grown to the L3/L4 larvae stage and infected by feeding for 2 h on YPD agar plates seeded with *C. albicans* SC5314. After infection, worms were washed several times with ddH_2_O and were finally resuspended in growth medium (3 g/L KH_2_PO_4_, 6 g/L Na_2_HPO_4_, 5 g/L NaCl, supplemented with 1.25 mM MgSO_4_ 10 µg/mL cholesterol, 100 µg/mL kanamycin and 75 µg/mL ampicillin) to a final concentration of approximately 40 worms/1.5 mL. In parallel, larvae of the non-infected control were fed on NGM plates seeded with *E. coli* OP50 and underwent the same procedure as infected nematodes. 1.5 mL of the worm suspension were transferred into each well of a 24-well plate (Greiner Bio One, Austria), nematodes were immediately counted (t0) and treated with either LIA, the solvent DMSO (0.2%) or remained untreated (uninfected control). Incubation and infection were done at 25°C. Infected and uninfected nematodes were monitored daily with a stereomicroscope and living worms were counted over a period of five days post infection. Number of living nematodes at t0 was set to 100% and survival at each time point was expressed compared to t0.

### Statistical analyses

The number of independent experiments (n) is indicated in the figure legends of the corresponding graphs. Each independent experiment was performed with a separate clone in at least three technical replicates. The results of all technical replicates were averaged for each experiment. For human cell culture and *C. elegans*, (n) is defined as independent experimental runs.

Overall normal distribution of data was analyzed by Shapiro-Wilk test and/or visual inspection of QQ-plots of residuals. Homogeneity of variance was tested using the Brown-Forsythe test. For normally distributed data, comparisons between two or multiple groups were done via analysis of variance (ANOVA). Non-normally distributed data were tested by the non-parametric Kruskal-Wallis test. P-values for the human cell viability assay and the *C. elegans* infection model were calculated using a two-way ANOVA approach, followed by the Benjamini-Hochberg procedure for multiple comparisons. *C. elegans* data were matched by time point, sphericity was not assumed and the Geisser-Greenhouse correction was used. Non-parametric data from time-kill assays were analyzed using a Kruskal-Wallis test followed by the two-stage step-up method of Benjamini, Krieger and Yekutieli. Acute toxicity data were analyzed within each timepoint by one-way ANOVA including linear trends for normally distributed data. Non-normally distributed data were examined by Kruskal-Wallis test. All statistical analyses were performed using GraphPad Prism 8 software.

## Results

### A screen for antifungal properties of lichen compounds reveals several potential antimycotics

**Figure 1:**
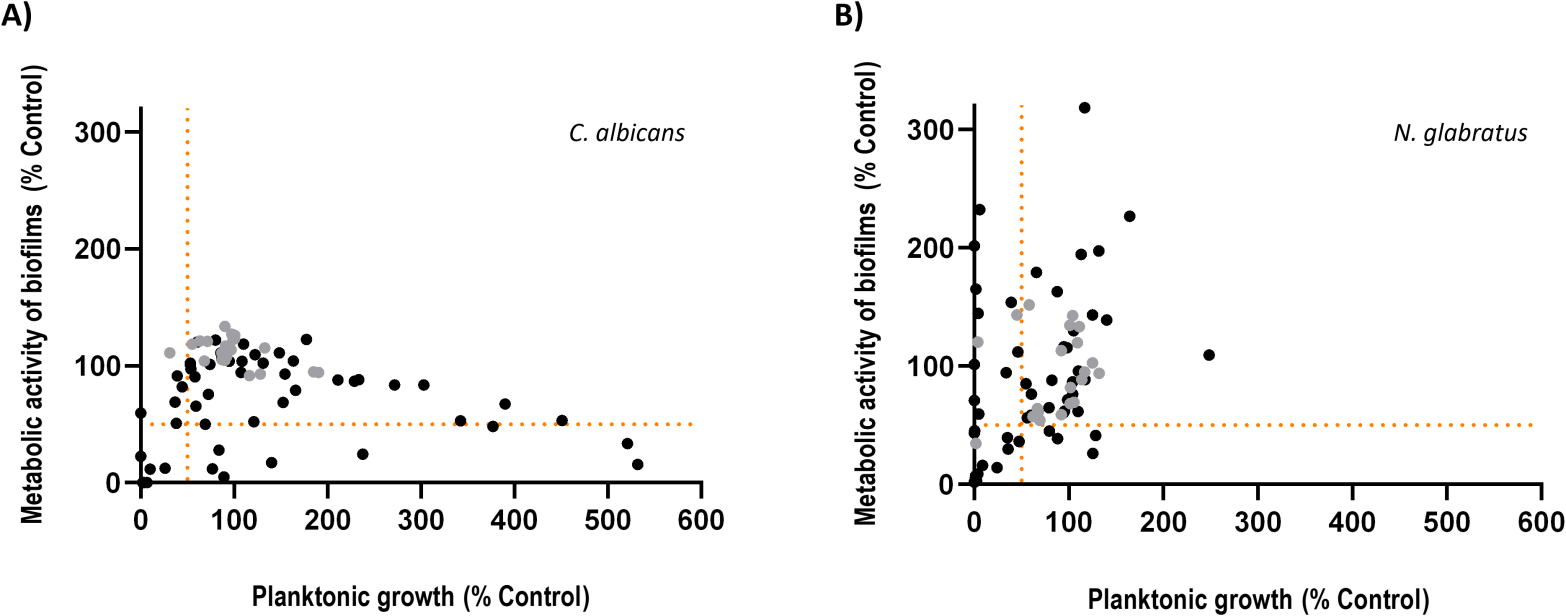
Several lichen-derived compounds exhibit inhibitory effects against *Candida albicans* and *Nakaseomyces glabratus* in planktonic growth and/or biofilms. *C*. *albicans***(A)** and *N. glabratus***(B)** were treated with lichen-derived compounds (LDC) and grown as planktonic cells or under biofilm-inducing conditions. Growth of planktonic cells was assessed by measurement of optical density at 490 nm. Inhibition of biofilm formation was assessed through measurement of metabolic activity by staining biofilms of *C*. *albicans* with the redox dye cell titer blue^®^ and biofilms of *N. glabratus* with the tetrazolium salt XTT. Measurements were normalized to untreated controls. Each data point represents one compound and shows the mean response of technical replicates (n=3) 24 h after treatment with the highest concentration tested for each compound. LDCs tested at 250 µM are depicted in black and data points depicted in grey show compounds which had to be tested at lower concentrations due to solubility. Inhibition of 50% or higher in any of the tested concentrations in either *C*. *albicans* or *N. glabratus* was set as the hit threshold and is depicted as orange-dotted lines.

Given the understudied yet promising antifungal potential of lichen-derived compounds (LDCs), we conducted a screen of 68 secondary metabolites, either directly isolated from the native lichen or synthesized based on known structural motifs (**Supplementary Table 1**). The screen was carried out in two clinically relevant species that differ significantly in virulence traits and resistance profiles: *C. albicans*, the major source of candidiasis, and *N. glabratus*, as a representative of NAC species. To evaluate antifungal efficacy, we (i) analyzed possible growth-inhibitory effects of LDCs in planktonic cells by measuring OD_490_ 24 h and 48 ± 1 h after LDC treatment. Biofilm formation is a strong determinant of fungal virulence and a driver of resistance. Thus, (ii) we parallelly assessed potential activity of the 68 LDCs against biofilm formation. For that purpose, we quantified metabolic activity under biofilm-inducing conditions 24 h after challenge with LDCs. All LDC treatments were performed in serial dilutions and compared with untreated controls. For these serial dilutions, the highest concentration was set at 250 µM; for compounds that were not completely soluble at the necessary stock concentration, the dilutions were started at lower concentrations (**Figure 1**, **Supplementary Table 1**).

The combined approach determining the impact on both growth and biofilm formation allowed for the evaluation of a broader antifungal potential. Accordingly, we defined our hits as such that would act against both planktonic cells and biofilms, yielding at least 50% inhibition in any of the tested concentrations in either *C. albicans* or *N. glabratus* (**Figure 1**). We set this cutoff, first, for comparability to published IC_50_ values and, second, following signal-to-noise considerations, *i.e*. a threshold stringent enough to minimize false positives and sufficiently sensitive to capture compounds with moderate but genuine activity that could be optimized through medicinal chemistry. Of note, previous screening campaigns have also specifically used 50% inhibition as their hit cutoff (Vila and Lopez-Ribot, 2016). Applying these criteria, our study yielded a total of 12 different hit LDCs. Five compounds (nos. 14, 23, 34, 37, 48) exhibited double inhibitory effects against planktonic growth and biofilm formation in *C. albicans* and ten (nos. 14, 21, 22, 34, 37, 47, 51, 56, 59, 60) in *N. glabratus*.

### Paraconic acids exhibit consistent anti-planktonic and anti-biofilm effects

**Figure 2:**
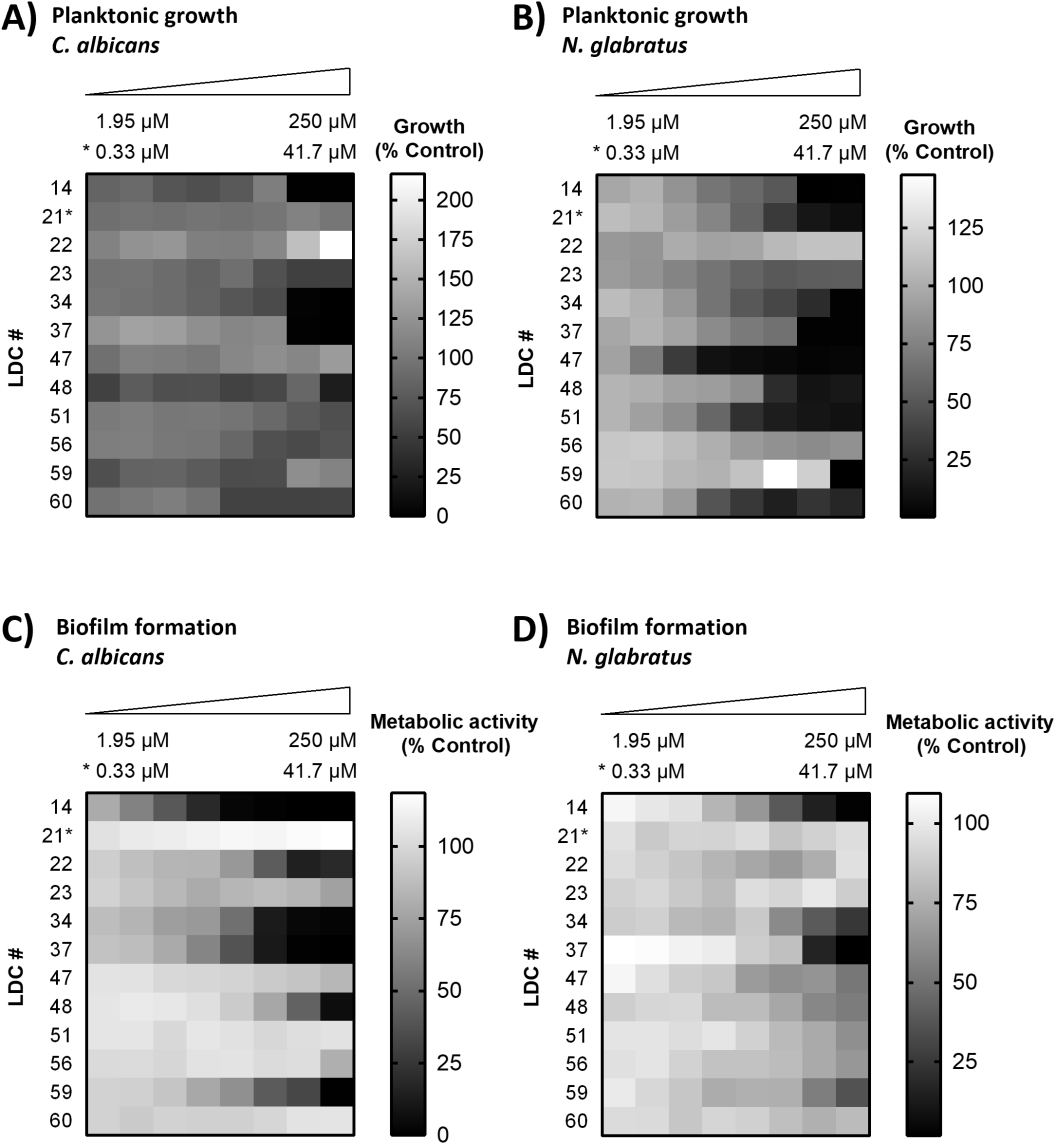
Validation of hit compound inhibitory activity 24 h post-treatment. Planktonic growth was determined in *C*. *albicans***(A)** and *N. glabratus***(B)** by measurement of OD_490_ 24 h after treatment with lichen-derived compounds (LDC) and Inhibition of biofilm formation in *C*. *albicans***(C)** and *N. glabratus***(D)** was assessed through measurement of metabolic activity by staining biofilms of *C*. *albicans* with the redox dye cell titer blue^®^ and biofilms of *N. glabratus* with the tetrazolium salt XTT. Measured values were normalized to untreated controls. LDC nos. 23 and 56 did not meet the established inhibition threshold of 50% after 24h for any assay. Data represent means of independent experiments, each performed with a separate clone (n=3). *, compounds with different concentration row due to solubility.

To confirm and further investigate the findings from the initial screen (**Figure 1**), growth and biofilm inhibition assays were conducted for all hit compounds using a low-throughput approach to generate dose-response curves. From these assays, we determined the minimum concentration required to inhibit 50% of growth (MIC_50_) and biofilm formation (BIC_50_) for *C. albicans* and *N. glabratus*, respectively (**Supplementary Table 2**). One LDC (no. 56) did not meet the established inhibition threshold of 50% for any assay and could thus not be validated as a hit. For four LDCs (nos. 14, 34, 37 and 48), we observed inhibition of planktonic growth of *C. albicans* below 50% (**Figure 2A**) with MIC_50_ values ranging between 65.1 µM and 239.0 µM after 24 h. One LDC (no. 23) reduced growth below 50% only after 48 h (**Supplementary Figure 1**). For nine LDCs (nos. 14, 21, 34, 37, 47, 48, 51, 59 and 60), we observed inhibition of planktonic growth of *N. glabratus* below 50% (**Figure 2B**) with MIC_50_ values between 5.9 µM and 177.9 µM after 24 h. Inhibition of *C. albicans* biofilm formation (**Figure 2C**) below 50% was observed for six LDCs (nos. 14, 22, 34, 37, 48 and 59) with BIC_50_ values between 5.7 µM and 115.6 µM, while formation of *N. glabratus* biofilms (**Figure 2D**) below 50% was observed upon treatment with four LDCs (nos. 14, 34, 37 and 59), obtaining BIC_50_ values between 45.9 µM and 153.5 µM.

Notably, the dibenzofurane (-)-isousnic acid (no. 34) and the two tested paraconic acids, (+)-lichesterinic acid (LIA, no. 37) and (-)-allo-proto-lichesterinic acid (aPLA, no. 14), were the only compounds that inhibited both growth and biofilm formation in both *C. albicans* and *N. glabratus*. Owing to their broad-spectrum activity, we prioritized these compounds as our top hits. While isousnic acid has already been associated with antifungal activity in previous studies (Goel et al., 2011a), LIA and aPLA remain poorly characterized in this context. Thus, we decided to continue our analyses only with LIA and aPLA (**Supplementary Figure 2**).

### (+)-Lichesterinic acid maintains moderate compatibility with human cells

**Figure 3:**
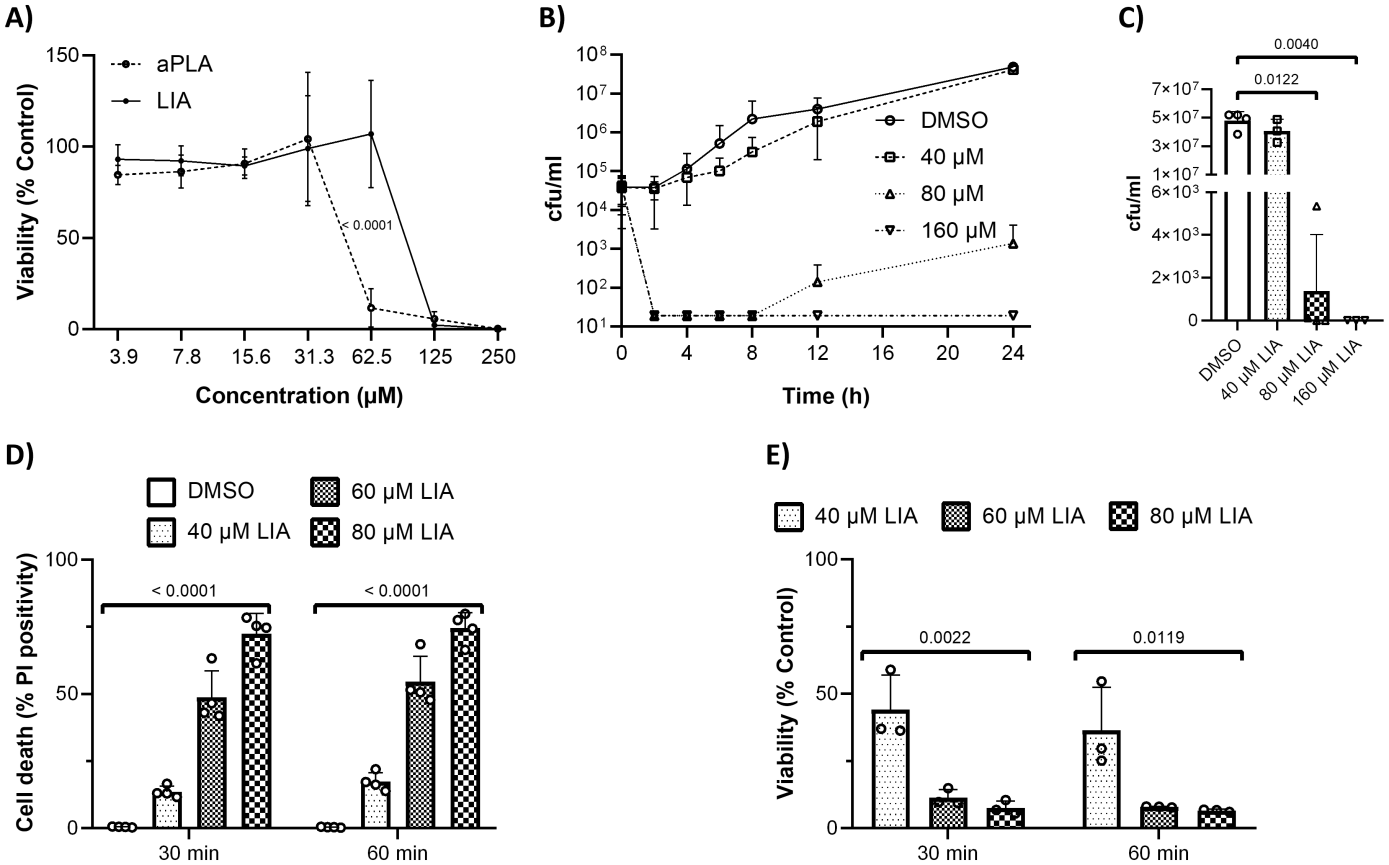
(+)-Lichesterinic acid exhibits moderate compatibility with human cells while exerting a fungicidal effect in *Candida albicans*. **(A)** Normal Human Dermal Fibroblasts were treated with serial dilutions of (-)-allo-Proto lichesterinic acid (aPLA) and (+)-lichesterinic acid (LIA). Viability was determined after 24 h of incubation by addition of CellTiter-Glo-Reagent and measurement of resulting luminescence. Measured values were normalized to controls treated only with the solvent (DMSO). Data represent means ± standard deviation (SD) of independent experimental runs (n=4). Statistical analysis was performed using two-way ANOVA followed by the Benjamini-Hochberg procedure for multiple comparison correction. Adjusted *p*-values are shown for significant differences (*p* < 0.05). **(B, C)***C*. *albicans* cultures were grown in RPMI while treated with different concentrations of LIA for 24 h. The cultures were sampled at the indicated timepoints **(B)** for colony count determination. Data show means ± SD of independent experiments, each performed with a separate clone (n=3-4). Statistical analysis was performed for the 24 h timepoint **(C)** using Kruskal-Wallis test followed by the two-stage step-up method of Benjamini, Krieger and Yekutieli for multiple comparison correction. Individual *p*-values are shown for significant differences (*p* < 0.05). **(D, E)** Cultures of *C*. *albicans* were grown to early logarithmic phase in YPD and then treated with different concentrations of LIA and sampled at the indicated timepoints. Cell death **(D)** was assessed by propidium iodide (PI) staining and analyzed by flow cytometry. Data show means + SD of independent experiments, each performed with a separate clone (n=4). Statistical analysis was performed using one-way ANOVA within each timepoint. *P*-values are shown for significant differences (*p* < 0.05). Survival **(E)** was determined via colony counting. Cfu/ml were calculated and normalized to untreated controls. Data show means + SD of independent experiments, each performed with a separate clone (n=3). Statistical analysis was performed within each timepoint using one-way ANOVA (for 30 min) or Kruskal-Wallis test (for 60 min). *P*-values are shown for significant differences (*p* < 0.05). Normal distribution of data was analyzed using Shapiro-Wilk test and visual inspection of QQ-plots of residuals. Homogeneity of variance was tested using Brown-Forsythe test. For more information on statistical methods used in **(A, C–E)** see Statistical analyses in Materials and Methods.

In order to evaluate the potential of LIA and aPLA for therapeutic applications, we first investigated their possible toxicity against human cells. In order to more accurately mimic the behavior of primary human cells, we chose non-immortalized cells, specifically Normal Human Dermal Fibroblasts (NHDF). After treatment with LIA and aPLA for 24 h, we assessed viability using a 2D-cell viability assay, which quantifies ATP to indicate the presence of metabolically active cells (**Figure 3A**). LIA was more tolerable than aPLA with an IC_50_ of 104.8 ± 7.5 µM (in comparison to 51.6 ± 9.9 µM for aPLA). Given the better overall tolerability of LIA, we focused our further efforts on this paraconic acid.

### (+)-Lichesterinic acid exerts fungicidal effects

We next assessed whether the antifungal effects of LIA derive from growth inhibition (fungistatic effects) or death induction (fungicidal effects). This distinction is crucial from a therapeutic point of view, since especially immunocompromised patients may not effectively eliminate fungal pathogens with fungistatic therapy alone. For that purpose, we conducted time-kill-assays in *C. albicans*, thus exposing cells to different concentrations of LIA, and counting viable colony-forming units (CFUs) at multiple time points during 24 h to assess changes in fungal burden (**Figures 3B, C**). While 40 µM did not significantly reduce viable cells over time, treatments with 80 µM and 160 µM resulted in complete killing of *C. albicans*. We also controlled for antifungal carryover, i.e. the residual presence of LIA upon CFU plating, which could have artificially suppressed fungal growth. However, we could not detect any carryover for any of the concentrations tested, suggesting a *bona fide* fungicidal effect of LIA (**Supplementary Figure 3**).

In addition to the standard time-kill experiments, we assessed the onset of antifungal activity in a logarithmically growing *C. albicans* culture by performing acute toxicity assays. Cultures were treated with LIA at concentrations of 40 µM, 60 µM, or 80 µM (corresponding to LIA’s MIC_50_). Higher concentrations were excluded as they eliminated all viable colonies in time-kill assays. Effects were assessed at 30- and 60 minutes post-treatment using two complementary methods: propidium iodide (PI) staining to detect dead cells (**Figure 3D**) and CFU counts normalized to DMSO-treated controls to evaluate viability (**Figure 3E**). Treatment with LIA resulted in a dose-dependent increase (*P*-value for trend = <0.0001 for 30 min and <0.0001 for 60 min) in PI-positive cells that was accompanied by a corresponding dose-dependent decrease in cell viability (*P*-value for trend = 0.0012 for 30 min; trend could not be assessed for 60 min as data was not normally distributed), suggesting strong effectivity in rapidly growing *C. albicans* cultures (**Figure 3**).

### (+)-Lichesterinic acid eradicates established biofilms in *C. albicans* and non-albicans species

**Figure 4:**
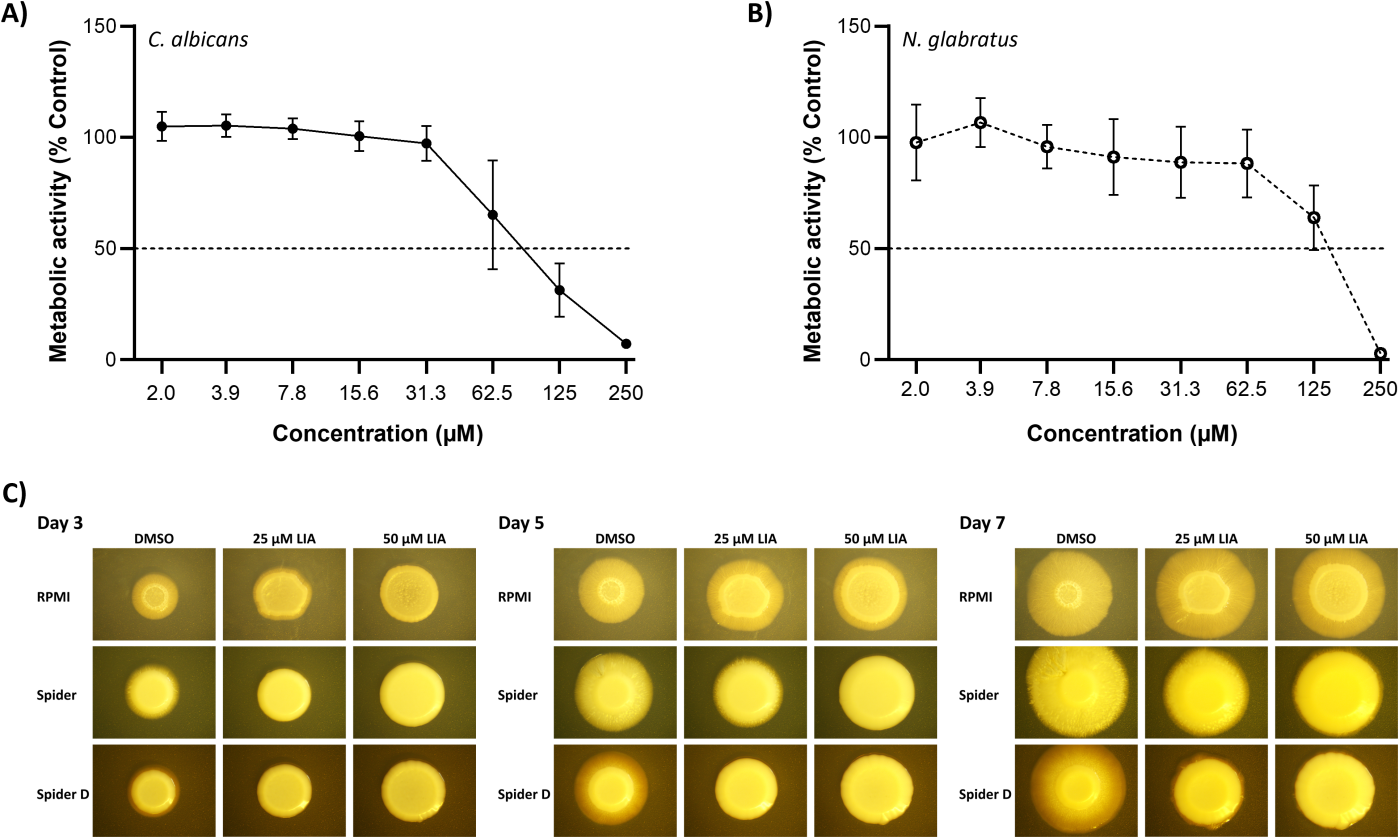
(+)-Lichesterinic acid exhibits anti-biofilm effects. Pre-grown mature biofilms of *C*. *albicans***(A)** and *N. glabratus***(B)** were treated with (+)-lichesterinic acid. Viability of biofilms was determined by assessing metabolic activity with CTB (*C. albicans*) or XTT (*N. glabratus*) staining 24 h after treatment. Measured values were normalized to untreated controls. Data represent mean ± SD of independent experiments, each performed with a separate clone (n=3). **(C)***C. albicans* was spotted onto different agar plates containing either LIA or its solvent only (DMSO) to evaluate filamentation on solid media. Images are representative of three independent experiments.

Biofilms remain a key challenge in antifungal therapy (Fan et al., 2022). As already assessed (**Figure 2**, **Supplementary Table 2**), LIA is able to inhibit biofilm formation in *C. albicans* (BIC_50_ 22.3 ± 4.1 µM) and *N. glabratus* (BIC_50_ 90.2 ± 26.0 µM), a crucial step for fungal colonization and persistence. Of note, this is also the case in two other non-albicans species, *C. dubliniensis* and *P. kudriavzevii*, in which LIA not only inhibits planktonic growth (**Supplementary Figure 4**) but also biofilm formation. However, the eradication of established biofilms represents a different major obstacle in effective antifungal treatment, since mature biofilms exhibit increased resistance to antifungal agents and protect embedded cells from host immune responses. Thus, we investigated whether LIA is able to eliminate pre-grown *C. albicans*, *N. glabratus, C. dubliniensis* and *P. kudriavzevii* biofilms by assessing mature biofilm metabolic activity. LIA was able to effectively eradicate biofilms of all tested pathogenic fungi with BEC_50_ values of 86.3 ± 26.8 μM in *C. albicans* (**Figure 4A**), 149.2 ± 25.6 µM in *N. glabratus* (**Figure 4B**), 189.7 ± 13.7 µM in *C. dubliniensis* (**Supplementary Figure 4C**) and 188.9 ± 12.3 µM in *P. kudriavzevii* (**Supplementary Figure 4F**), respectively. Altogether, LIA is effective in both preventing biofilm formation and disrupting mature biofilms.

### (+)-Lichesterinic acid impairs filamentation of *C. albicans*

In *C. albicans*, filamentation processes involving the formation of true hyphae or robust pseudohyphae play a major role in biofilm formation and virulence (in contrast, *N. glabratus* does not undergo true filamentation under standard conditions) (Galocha et al., 2019). Thus, we investigated the effects of LIA on *C. albicans* filamentation using different media. Colonies were grown on standard RPMI or filamentation-inducing (Spider and Spider D) agar plates supplemented with either 25 µM or 50 µM LIA (corresponding to the 1x and 2x BIC_50_ values observed *in vitro*), and filamentation was monitored over 7 days; DMSO-only plates served as controls. On RPMI plates, LIA demonstrated dose-dependent inhibition of filamentation with the strongest effects apparent at day 3 (**Figure 4C**; **Supplementary Figure 5**). Although filamentation increased by day 7 in treated samples, clear differences between treatments and controls persisted. Spider plates revealed more dramatic inhibitory effects, with both 25 µM and 50 µM LIA treatments completely preventing radial filamentation at day 3 and allowing only for minimal filamentation at later days (**Figure 4C**; **Supplementary Figure 5**). These inhibitory effects were even further exacerbated in Spider D plates (**Figure 4C**; **Supplementary Figure 5**), in which mannitol is replaced by a lower concentration of glucose. These results provide a qualitative demonstration that LIA effectively inhibits *C. albicans* filamentation in a dose- and media-dependent manner, with the strongest effects observed under conditions where filamentation is actively promoted.

### (+)-Lichesterinic acid promotes survival *in vivo* in a *Caenorhabditis elegans* fungal infection model

**Figure 5:**
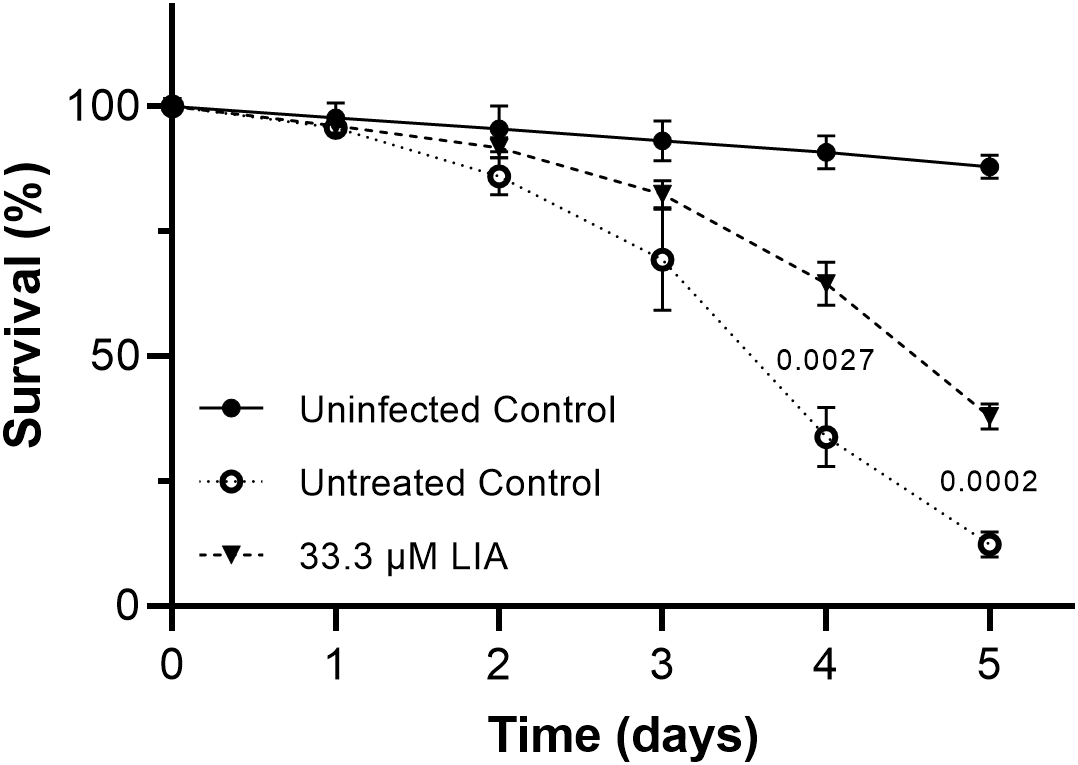
(+)-Lichesterinic acid promotes survival of *Caenorhabditis elegans* infected with *Candida albicans*. Nematodes in L3 and L4 stage were infected with *C. albicans*, transferred to liquid M9 growth media and treated with LIA. Survival was monitored daily under a stereomicroscope based on classical features like movement and responsiveness. Data represent mean ± SD of independent experimental runs (n=3). Normal distribution of data was confirmed by Shapiro-Wilk test and homogeneity of variance by the Brown-Forsythe test. Statistical analysis was performed using two-way ANOVA with Geisser-Greenhouse correction followed by the Benjamini-Hochberg procedure for multiple comparison (see Statistical analyses in Material and Methods). Adjusted *p*-values are shown for significant differences (*p* < 0.05) between LIA-treated worms and untreated controls (days 4 and 5 post-infection).

Following our combined *in vitro* results, we finally decided to evaluate the antifungal efficacy of LIA *in vivo*. For that purpose, we employed the nematode *Caenorhabditis elegans*, which has been proven to be a valuable infection model, for instance, for the identification of virulence factors and the evaluation of antifungal agents (Breger et al., 2007; Delattin et al., 2014). Preliminary testing revealed that LIA concentrations of 50 µM and above were toxic to *C. elegans* (**Supplementary Figure 6**), thus we proceeded with lower concentrations. In brief, worms synchronized in late larval stage were infected with *C. albicans* and treated with LIA. Animals were monitored daily over five days, with live and dead animals scored under a stereomicroscope. After 5 days of incubation, uninfected control worms showed 87.9% mean survival, while infected, untreated worms exhibited only 12.3% survival. Treatment with 33.3 µM LIA significantly increased survival rates to 38% (**Figure 5**). These results demonstrate that LIA provides protective effects against *C. albicans* infection at concentrations tolerated by the host organism.

## Discussion

Treatment of biofilm-related *Candida* infections is challenging due to reduced susceptibility to common antifungals like fluconazole (Czajka et al., 2023). The use of alternative treatments such as echinocandins or lipid formulations of amphotericin B is well established, but may carry disadvantages like the lack of oral formulations and additional risks (like low nephrotoxicity risk for liposomal amphotericin B) (Nivoix et al., 2020). In any case, the treatment of biofilms generally requires substantially higher doses and prolonged use of a given antifungal compound, increasing both the overall costs and potential toxicity. Thus, finding novel compounds with the capacity to inhibit the formation and/or eradicate pre-grown fungal biofilms is critical to enhance current antifungal therapies and ensure the efficacy of future treatments against FIs. In this report, we identify and characterize the antibiofilm activity of LIA, which emerged as a top hit upon screening lichen secondary metabolites for antifungal activity. LIA reduced biofilm formation and growth of planktonic cells in *C. albicans* and different NAC, showed fungicidal effects and also compromised filamentation in *C. albicans*. Importantly, LIA was not only able to inhibit biofilm formation, but also eradicated established biofilms. This is a meaningful and distinctive characteristic, since mature biofilms, once fully developed, are significantly more challenging to treat and eliminate with most antifungals (Pierce et al., 2015). Furthermore, LIA significantly improved survival of nematodes in an *in vivo* infection model of *C. elegans* at a dose tolerable by the host.

LIA is a paraconic acid, which belongs to the chemical class of aliphatic lactones and can be found in various lichen species including *Cetraria islandica* (Sánchez et al., 2022). It has been associated with inhibition of 5-lypoxygenase (Ingolfsdottir et al., 1994), inhibition of pigmentation (Abrahams et al., 2008) and anti-trypanosomal activity (Igoli et al., 2014). Interestingly, a related paraconic acid, aPLA, also showed consistent antifungal effects in our assays. While both of these compounds compromised viability of human NHDFs at higher concentrations, LIA was more tolerable than aPLA. Our *in vivo* studies in *C. elegans* demonstrated that at higher concentrations, LIA indeed exhibited partial toxicity. Nevertheless, we identified a therapeutic window at lower concentrations, in which no adverse effects were observed, while a significant (>3-fold) increase in survival upon infection was achieved. However, cell culture assays do not capture organ-level responses, metabolism, or pharmacokinetic handling, and *C. elegans* – while providing whole-organism context - differs substantially from mammals in relevant pharmacokinetic aspects and metabolic pathways. Thus, the observed therapeutic window remains provisional and needs corroboration in further preclinical models, including mammals. This does not only relate to efficacy studies, pharmacokinetic and pharmacodynamic properties, but – in light of the potential host toxicities we observe in *C. elegans* – also requires focused follow-up, including potential tissue-specific toxicity. Addressing these points will clarify LIA’s true clinical potential and inform rational go/no-go decisions.

In addition, reformulation efforts or testing of derivatives may help optimize the potential applicability of LIA. Mechanistic studies to determine whether the observed fungicidal effects arise from on-target effects in host cells, off-target binding, or toxic metabolites will be essential to guide optimization. Notably, multiple synthetic routes have been devised for paraconic acids, including the total synthesis of (±)‐lichesterinic acid (Fernandes et al., 2020), which may serve as a basis to generate and explore other LIA derivatives for enhanced antifungal activities and/or increased safety margins.

While for LIA and aPLA there are no reports on antifungal activities, a related stereoisomer, protolichesterinic acid (PLA) has been previously shown to counteract growth of diverse phytopathogens (Goel et al., 2011b). In addition, PLA also exerts growth-inhibitory effects in clinically relevant species, including *C. albicans*, *C. tropicalis*, *Aspergillus flavus*, and *Trichophyton rubrum* (Sasidharan et al., 2014) and has been shown to reduce pathogenicity of *C. albicans* by inhibiting the H_2_S-synthesizing cystathionine synthase and reduce fungal burden in a murine candidiasis model (Chang et al., 2022). However, to our knowledge, possible effects of PLA on fungal biofilms have not been analyzed and would be an interesting point to inspect in the future. Of note, our screen also revealed a related aliphatic acid, norrangiformic acid, with (more moderate) growth-inhibitory effects on both *C. albicans* and *N. glabratus.* However, we did not observe any potential to inhibit biofilms. While norrangiformic acid alone has not been directly connected to antifungal effects yet, lichen extracts containing this metabolite (along others) have been reported to inhibit growth of *C. albicans* and *N. glabratus* (Brakni et al., 2018).

Intriguingly, a number of LDCs have demonstrated antifungal activity, including growth-inhibitory effects against fungal pathogens from *Candida* spp. and *Aspergillus* spp (Poulsen-Silva et al., 2025). For instance, several depsides, including atranorin (Ranković et al., 2014), chloroatranorin (Türk et al., 2006) or gyrophoric acid (Candan et al., 2006) as well as depsidones like lobaric (Thadhani et al., 2012), evernic and physodic acid (Kosanić et al., 2013) have been shown to inhibit growth of several *Aspergillus* and *Candida* species, including *C. albicans*. Among metabolites from other chemical classes, the dibenzofuran usnic acid has been consistently associated to antifungal activities (Pires et al., 2012; Ranković et al., 2014), including anti-biofilm properties in *C. albicans* (Peralta et al., 2017). The herein presented screen also included usnic acid, which showed growth-inhibitory effects against planktonic *N. glabratus* (although not against *C. albicans*). While in our hands, usnic acid did not inhibit biofilm formation, this does not exclude the possibility that it does eradicate pre-formed biofilms in *C. albicans* as has been previously reported (Peralta et al., 2017). In our screen, we also tested the usnic acid isomer, (-) isousnic acid (no. 34), just differing by two interchanged substituents, which interestingly, did show biofilm-inhibitory effects in *C. albicans* and *N. glabratus*. Isousnic acid also inhibited planktonic growth and biofilm formation of both *C. albicans* and *N. glabratus*. To our knowledge, isousnic acid has only been related to inhibition of plant pathogenic fungi so far (Goel et al., 2011a). Finally, another dibenzofurane, didymic acid (no. 22), also showed antifungal effects, albeit they were limited to the inhibition of biofilm formation of *C. albicans*.

Besides the mentioned paraconic acids and dibenzofurans, our screen revealed further LDCs surpassing our established 50%-inhibition threshold, although the effects were either restricted to one of the two lifecycle modalities (planktonic or biofilm) and/or to one of the two tested species (*C. albicans* or *N. glabratus*) and/or showed smaller effect sizes than the top hits isousnic acid, LIA and aPLA (**Table 1**). For instance, the depside sphaerophorin (no. 59) showed moderate inhibition of planktonic growth of *N. glabratus* and biofilm formation of both tested species. To our knowledge, sphaerophorin has only been associated with antifungal properties against certain dermatophytes, mostly at relatively high concentrations (Furmanek et al., 2019). The depsidone diploicin (no. 23), for instance, prevented only planktonic growth, showing no effect against biofilms. Of note, there are no previous reports on antifungal effects of diploicin. Another depsidone, stictic acid (no. 60), displayed anti-planctonic effects in *N. glabratus*; it has been attributed some effects against several fungal species (not *Candida* spp.) at rather high concentrations (Ranković and Mišić, 2008). In addition, stictic acid – as the anthraquinone parietin - has previously been suspected to be involved in the anti-maturation process of *C. albicans* biofilms as the major compounds of some active lichen extracts (Millot et al., 2017). The depsidones psoromic acid (no. 51), physodalic acid (no. 47) and cetraric acid (no. 21) showed strong effects against planktonic *N. glabratus* but not against *C. albicans*. For physodalic acid and psoromic acid, antifungal properties have not been reported, although complex lichen extracts containing these metabolites (alongside other compounds) show antifungal properties in different fungi (Mahadik et al., 2011; Furmanek et al., 2022). To our knowledge, there are no bioactivity reports on cetraric acid, which in our hands strongly inhibited planktonic growth of *N. glabratus*. Finally, the depsidone physodic acid (no. 48), for which antifungal properties have been previously reported (Kosanić et al., 2013), inhibited planktonic growth of both tested species and also prevented biofilm formation in *C. albicans*, but failed to do so in *N. glabratus*. Note that most of the triterpenoid compounds were tested at lower concentrations due to their poor solubility in the culture medium. Thus, triterpenoids not reaching the established threshold might still be candidates for future studies using alternative application strategies.

**Table 1:** Antifungal properties of lichen-derived compounds tested in this study.

Name	Class	*C. albicans* (Plnk)	*C. albicans* (BioF)	*N. glabratus* (PlnK)	*N. glabratus* (BioF)	Reports
allo-Proto lichesterinic acid	Paraconic acid	✓	✓	✓	✓	This study
Cetraric acid	Depsidone	X	X	✓	X	This study
Didymic acid	Dibenzofurane	X	✓	X	X	This study
Diploicin	Depsidone	✓	X	X	X	This study
Isousnic acid	Dibenzofurane	✓	✓	✓	✓	Goel et al., 2011b
Lichesterinic acid	Paraconic acid	✓	✓	✓	✓	This study
Physodalic acid	Depsidone	X	X	✓	X	This study
Physodic acid	Depsidone	✓	✓	✓	X	Kosanić et al., 2013
Psoromic acid	Depsidone	X	X	✓	X	This study
Sphaerophorin	Depside (Didepside)	X	✓	✓	✓	Piovano et al., 2002
Stictic acid	Depsidone	X	X	✓	X	Ranković and Mišić, 2008

Only hit compounds of the initial screen that could be subsequently validated to yield at least 50% inhibition in any of the applied low throughput assays are shown. For several compounds, antifungal activity has been reported previously; the corresponding references listed include only studies that examined the antifungal effects of the pure compounds, not crude extracts. PlnK, inhibition of planktonic (Plnk) growth; BioF, inhibition of biofilm (BioF) formation; ✓, corresponding inhibition was above the threshold of 50% in this study; X, no inhibition or inhibition below 50% in this study.

Altogether, the present study adds to mounting evidence, reporting LDCs as a potential source for novel antifungal agents. However, research specifically examining anti-biofilm properties of LDCs against *Candida* spp. remains very limited. To date, only evernic acid (Girardot et al., 2021) and usnic acid (Nithyanand et al., 2015) have shown potential in eradicating preformed biofilms, while retigeric acid B has been reported to exhibit synergistic effects with azoles in inhibiting biofilm formation in *C. albicans* and NAC species (Chang et al., 2012). Our study identifies a series of further LDCs with anti-biofilm potential and highlights the paraconic acid LIA as an LDC with strong multifaceted antifungal activity. LIA is not only effective against planktonic cells, but also inhibits biofilm formation and promotes mature biofilm eradication. These activities are present in *C. albicans* as well as in the NAC species *N. glabratus, C. dubliniensis* and *P. kudriavzevii*, underlining the broad-spectrum activity of LIA. Our cytotoxicity data in human cells, together with our *in vivo* studies in *C. elegans*, suggest that while LIA displays some toxicity at elevated concentrations, a defined therapeutic window is evident at lower concentrations. This window, in which antifungal efficacy is achieved without overt adverse effects, highlights the potential of LIA as a promising candidate for further preclinical evaluation.

## Data Availability

The raw data supporting the conclusions of this article will be made available by the authors, without undue reservation.
