# Supplementary material for: The lichen secondary metabolite lichesterinic acid exhibits antibiofilm activity against fungal pathogens
Source: Front Cell Infect Microbiol. 2026 Jan 12;15:1730365. doi: 10.3389/fcimb.2025.1730365 (PMC12832668; doi:10.3389/fcimb.2025.1730365)
Supplement: Supplementary file 1 [file Image1.pdf]

Supplemental Figures for

The lichen secondary metabolite lichesterinic acid exhibits antibiofilm activity against fungal pathogens

Odabas et al.

Contents

Supplemental Figures 1-6

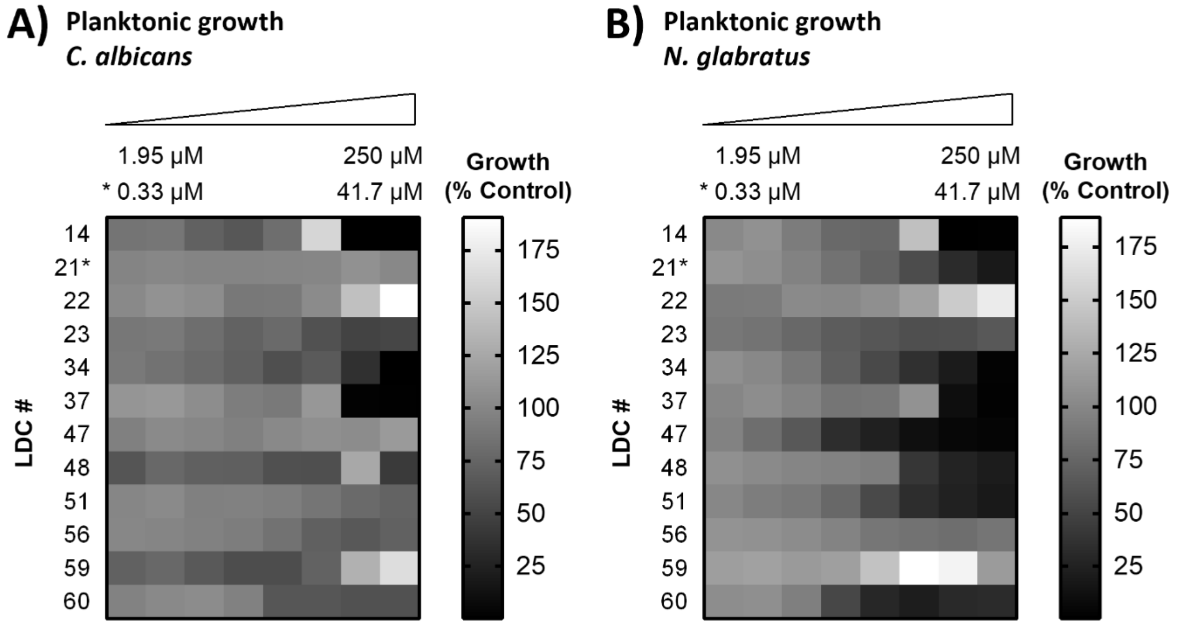

Supplemental Figure 1, related to Figure 2. Validation of hit compound inhibitory activity 48h post-treatment. Planktonic growth was determined in *C. albicans* **(A)** and *N. glabratus* **(B)** by measurement of OD_{490} 48 h after treatment with lichen-derived compounds (LDC). Measured values were normalized to untreated controls. Inhibition-threshold was set at 50%. Data represent means of independent experiments, each performed with a separate clone (n=3). *, compounds with different concentration row due to solubility.

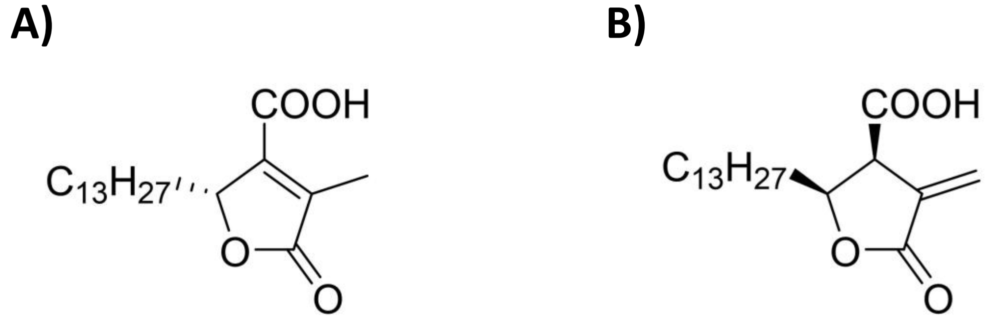

Supplemental Figure 2. Chemical structures of the paraconic acids (+)-lichesterinic acid (A) and (-)-allo-proto-lichesterinic acid (B).

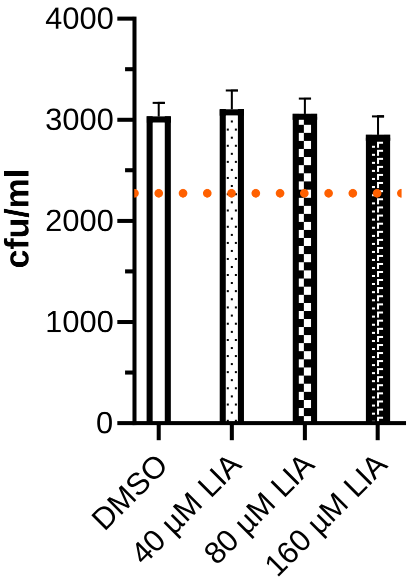

Supplemental Figure 3, related to Figures 3B and 3C. (+)-Lichesterinic acid does not show antifungal carryover in a time-kill assay. Antifungal carryover was assayed for all (+)-lichesterinic acid (LIA) treatment concentrations of the time kill assay. *C. albicans* was treated and then immediately plated onto YPD agar plates. After incubation, colonies were counted and cfu/ml were calculated. Data show mean + SD of one experiment (*n*=3). Antifungal carryover was considered absent when compound treatment did not exceed a difference of 25%. cfu/ml compared to the solvent (DMSO)-only treated controls. The 25% threshold is indicated by the dotted orange line, which was not surpassed upon LIA treatment.

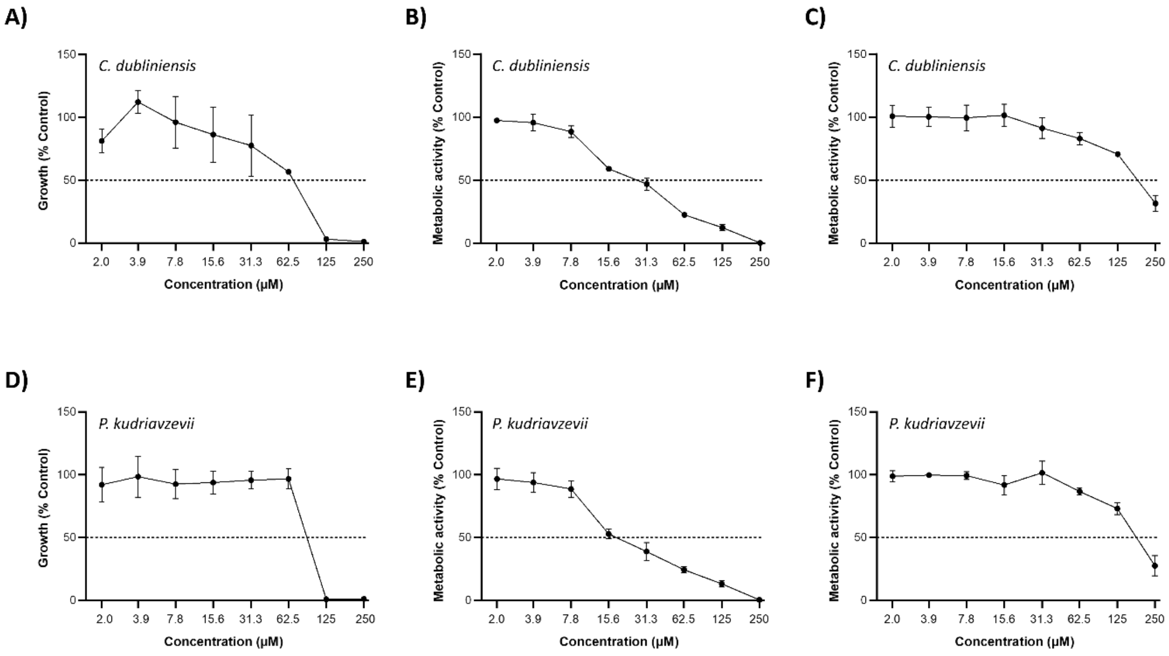

Supplemental Figure 4. (+)-Lichesterinic acid exhibits anti-planktonic and anti-biofilm effects in *Candida dubliniensis* and *Pichia kudriavzevii*. *C. dubliniensis* **(A)** and *P. kudriavzevii* **(D)** planktonic growth, *C. dubliniensis* **(B)** and *P. kudriavzevii* **(E)** cells grown under biofilm-inducing conditions, and *C. dubliniensis* **(C)** and *P. kudriavzevii* **(F)** pre-grown mature biofilms were treated with serial-dilutions of (+)-lichesterinic acid. Growth of planktonic cells was assessed by measurement of optical density at 490 nm 24 h after treatment. Inhibition of biofilm formation and biofilm eradication were assessed through measurement of metabolic activity by staining biofilms with the tetrazolium salt XTT 24 h after treatment. All measured values were normalized to untreated controls. Data represent means ± SD of independent experiments, each performed with a separate clone (n=3).

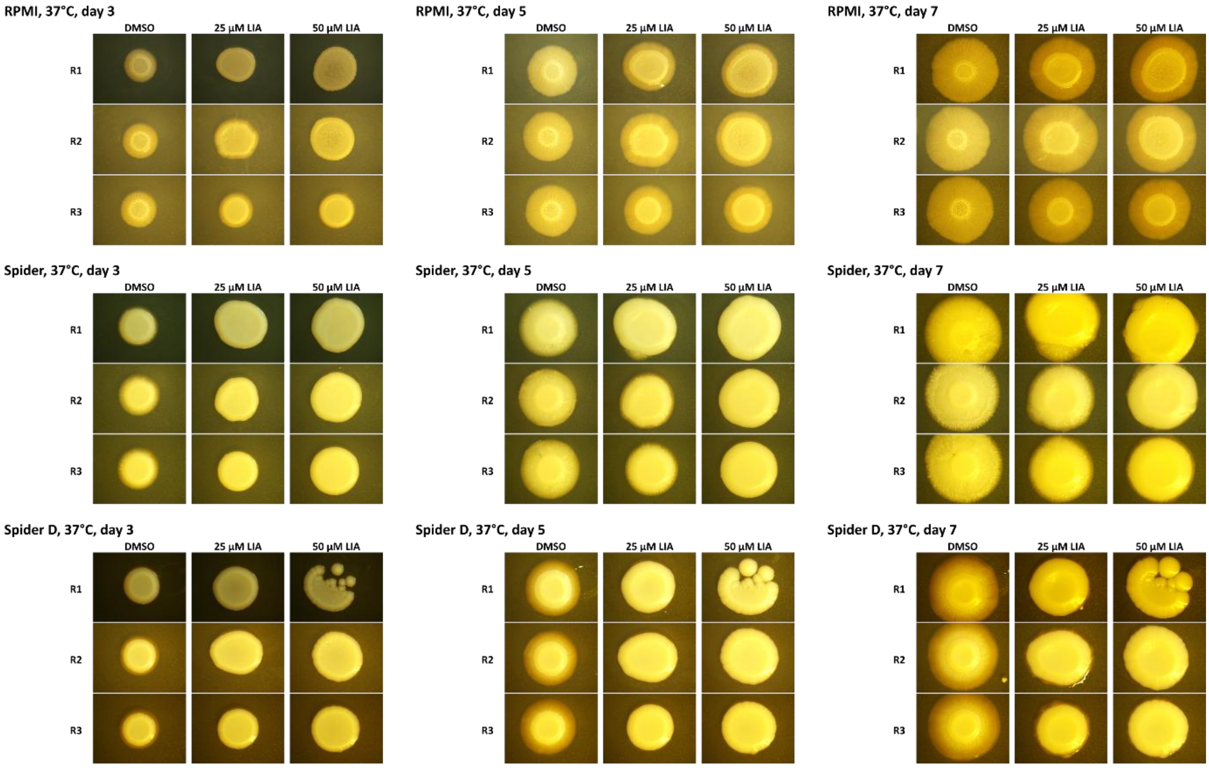

Supplemental Figure 5, related to Figure 4C. (+)-Lichesterinic acid inhibits filamentation in a solid filamentation assay. *Candida albicans* cells were spotted onto different media agar plates containing either 25 µM, 50 µM (+)-lichesterinic acid (LIA) or only its solvent (DMSO).

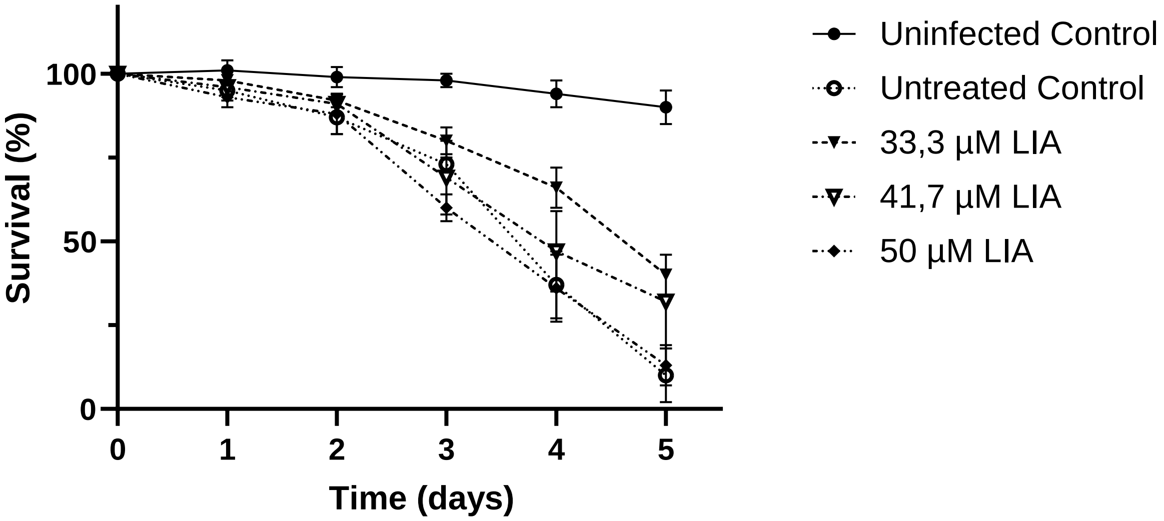

Supplemental Figure 6, related to Figure 5. Low doses of (+)-lichesterinic acid promote survival of *Caenorhabditis elegans* infected with *Candida albicans* while higher doses lose the protective effects. Nematodes in L3 and L4 stage were infected with *C. albicans*, transferred to liquid M9 growth media and treated with (+)-lichesterinic acid (LIA). Survival was monitored daily under a stereomicroscope based on classical features like movement and responsiveness. Data represent means ± SD of one experiment (*n*=3-4).
